# PVA/Chitosan/Silver Nanoparticles Electrospun Nanocomposites: Molecular Relaxations Investigated by Modern Broadband Dielectric Spectroscopy

**DOI:** 10.3390/nano8110888

**Published:** 2018-11-01

**Authors:** Mohammad K. Hassan, Ahmed Abukmail, Alaa J. Hassiba, Kenneth A. Mauritz, Ahmed A. Elzatahry

**Affiliations:** 1 Center for Advanced Materials, Qatar University, Doha 2713, Qatar; mohamed.hassan@qu.edu.qa; 2 Department of Computing Sciences, University of Houston—Clear Lake, Houston, TX 77058, USA; Abukmail@UHCL.edu; 3 Materials Science & Technology Program, College of Arts & Sciences, Qatar University, Doha 2713, Qatar; ala.hasb@gmail.com; 4 School of Polymers and High Performance Materials, The University of Southern Mississippi, Hattiesburg, MS 39406, USA; Kenneth.Mauritz@usm.edu

**Keywords:** electrospinning, chitosan, poly (vinyl alcohol), nanofibers, silver nanoparticles, dielectric spectroscopy

## Abstract

In this study, we used broadband dielectric spectroscopy to analyze polymer nanofibers of poly(vinyl alcohol)/chitosan/silver nanoparticles. We also studied the effect of incorporating silver nanoparticles in the polymeric mat, on the chain motion dynamics and their interactions with chitosan nanofibers, and we calculated the activation energies of the sub-*T_g_* relaxation processes. Results revealed the existence of two sub-*T_g_* relaxations, the first gets activated at very low temperature (−90 °C) and accounts for motions of the side groups within the repeating unit such as –NH_2_, –OH, and –CH_2_OH in chitosan and poly(vinyl alcohol). The second process gets activated around −10 °C and it is thought to be related to the local main chain segments’ motions that are facilitated by fluctuations within the glycosidic bonds of chitosan. The activation energy for the chitosan/PVA/AgNPs nanocomposite nanofibers is much higher than that of the chitosan control film due to the presence of strong interactions between the amine groups and the silver nanoparticles. Kramers–Krönig integral transformation of the *ε*′′ vs. *f* spectra in the region of the chitosan *T_g_* helped resolve this relaxation and displayed the progress of its maxima with increasing temperature in the regular manner.

## 1. Introduction

Polymeric nanofibers have been implemented in many applications and different areas, including the medical field. The physical attributes of nanofibers, i.e., porous membranes and large surface area to volume ratios, make them good candidates for variety of applications, such as tissue engineering, wound dressings, drug delivery, energy, food packaging and biosensors [1,2]. Different methods have been used to create nanofibers, however, the electrospinning technique is the most well-known for its simplicity to produce fibers from different materials in large scale, high porosity and high surface area [2,3]. Formation of fibers is mainly a result of inducing charges in the polymer solution using high voltage power supply. This leads to generating a repulsive force leading to fiber formation after evaporation of the solvent [4]. Chitosan is one of the known polymers extensively used in biomedical applications due to its biocompatibility, biodegradability and antibacterial properties [5]. Chitosan’s chemical structure is characterized by the presence of an –NH2 group, which supports many functions including antibacterial activity, and acts as a reducing agent to enhance its solubility in water upon protonation in acidic medium [6,7]. Many factors have to be taken into consideration during the electrospinning process of chitosan including molecular weight, degree of deacetylation and degree of protonation [6,7]. On the other hand, chitosan has been incorporated with other polymers such as poly (vinyl alcohol) to produce electrospun fibers to enhance the fiber formation and morphology of produced fibers [8]. 

Recently, silver nanoparticles (AgNP) became an attractive option to many researchers due to their potential in several applications including biomedical applications such as wound dressing, antiviral drugs, and antibacterial and anti-inflammatory agents [9,10,11]. In recent years, nanocomposites were developed for such applications by incorporating AgNPs in polymers as the matrix material to allow ease of processing and handling. A candidate polymer that can be combined with silver nanoparticles is chitosan for its low cost, abundance, and for being an environmentally friendly polymer [12]. 

Although there has been dielectric (impedance) investigations of chitosan/AgNP films [12], to the best of our knowledge, this is the first study that involved the electrospun nanofibers of PVA/chitosan/AgNP nanocomposites. This article is a continuation of previously published work on the synthesis and antimicrobial properties of these nanocomposite fibers for wound dressing applications [13]. 

## 2. Experimental

### 2.1. Electrospinning Nanofiber Preparations

Details of sample preparation were reported in our previous work [13]. Typically, the PVA solution with a concentration of 8 wt/wt% was first prepared by dissolving the polymer in distilled water at 80 °C using magnetic stirrer, and then chitosan solution was prepared by dissolving it in 2 *v*/*v*% acetic acid at a temperature of 60 °C. PVA and chitosan solutions were then mixed at a concentration of 12/4.7 wt/wt% and stirred well to ensure homogeneous mixing. Finally, silver nitrate was added to the polymer mixture to create a PVA/chitosan/AgNO_3_ solution. It should be noted that adding 0.0198 g of silver nitrate yields 0.0126 g of silver. The electrospun fibers were obtained from the nanocomposite blend solution at 18 kV voltage and a 10 cm collection distance at a rate of 0.3 mL/h. The nanofibers films were stored in a desiccator until the dielectric spectroscopy experiments.

### 2.2. Dielectric Spectroscopy Measurements

Dielectric spectra were generated isothermally via a Novocontrol GmbH Concept 40 broadband dielectric spectrometer (BDS) (Novocontrol Technologies GmbH, Montabaur, Germany) over a temperature range of −90 to 200 °C and a frequency range of 0.1 Hz–3 MHz. Samples were stored in a desiccator at room temperature for about one week before BDS experiments were performed to reduce the obscuring effect of water on the dielectric response. Samples were covered on both sides with clean aluminum sheets. Then, the assembled sample was placed in the middle of two stainless steel electrodes with a diameter of 2 cm. Finally, samples were placed in the instrument for data collection. To avoid any possible spurious effects due to the measuring cell and to compensate for the long-term drift effects, the following calibrations were conducted before testing:

- Internal interface all calibration
- External interface low impedance load short calibration
- External low capacity open calibration

The Havriliak–Negami (H-N) equation [14,15,16], shown below, was fitted to the experimental data to obtain the dielectric parameters that are reflective of motional time scales and distributions of local structure on the distance scale of the relaxations:

(1) $$\varepsilon *\left(\omega \right)=\varepsilon^{\prime }-i\varepsilon^{\prime \prime }=-i{\left(\frac{\sigma_{dc}}{\varepsilon_{0}\omega }\right)}^{N}+\overset{3}{\underset{k=1}{\sum }}\left[\frac{\Delta \varepsilon_{k}}{{\left(1+{\left(i\omega \tau_{HN}\right)}^{\alpha_{HN}}\right)}^{\beta_{HN}}}+\varepsilon_{\infty k}\right]\;$$

where *ε*′ is the real dielectric permittivity; *ε*′′ is the corresponding imaginary dielectric permittivity at the same frequency; and *i* = √−1. The sum contains three relaxation terms while the left term is meant to account for dc conductivity, if present. *ε*_0_ is the vacuum permittivity, *ω* = 2*πf*, and *σ_dc_* represents dc conductivity. For each relaxation term *k*, the dielectric strength Δ*ε_k_ =* (*ε_R_* − *ε_∞_*)*_k_* is the change between *ε*′ at low and high frequencies, respectively. The exponent *N* is interpreted as reflecting the nature of charge hopping pathways and mobility constraints as described earlier [17]. $\alpha_{HN}$ and $\beta_{HN}$ are empirical parameters related to the symmetric and asymmetric broadening of the relaxation peak, respectively. The Havriliak–Negami relaxation time *τ_HN_* is related to the actual relaxation time *τ_max_* = (*f_max_*)^−1^ where *f_max_* is the frequency at loss peak maximum, by the following equation [18]:

(2) $$\tau_{\max}=\tau_{HN}{\left[\frac{\sin\left(\frac{\pi \cdot \alpha_{HN}\cdot \beta_{HN}}{2\left(\beta_{HN}+1\right)}\right)}{\sin\left(\frac{\pi \cdot \alpha_{HN}}{2\left(\beta_{HN}+1\right)}\right)}\right]}^{\frac{1}{\alpha_{HN}}}\;$$

In Equation (2), the dc term is attributed, in a general way, to unintended or inherent charge migration, which often obscures loss peaks so that removal of this feature is needed.

## 3. Results and Discussion

### 3.1. Morphology Study

**Figure 1:**
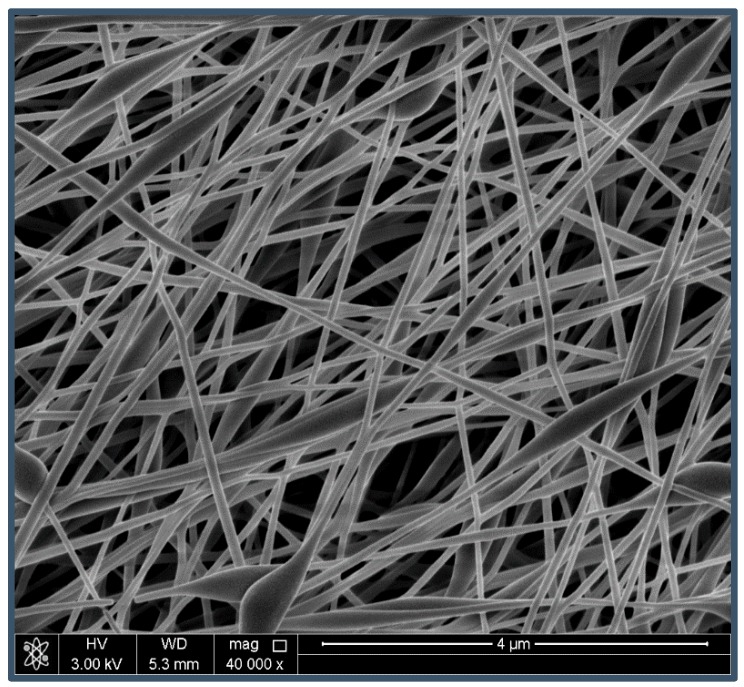
Scanning electron microscopy images at 40,000× of electrospun nanofibers of PVA/chitosan/AgNPs blends at PVA/Chitosan a concentration of 12/4.7 wt/wt%, respectively, using the electrospinning conditions of 10 cm, 18 kV, and 0.3 mL/h.

The scanning electron micrograph in Figure 1 presents the morphology of the PVA/chitosan/AgNP fibers electrospun, which showed a typical homogenous cylindrical-like structure with average fiber diameter of 200.0–250.0 nm and beads-on-string morphology. Previously, we reported the antimicrobial activities of the as-prepared nanofibrous mats as a result of loaded face-centered cubic structure nanosized silver particles [13].

### 3.2. Dielectric Measurements

Broadband dielectric spectroscopy (BDS) is a tool with great potential for material characterization as it can interrogate polymer chain motions over broad frequency (*f*) ranges, temperatures and, therefore, over a wide range of distance and time scales [18,19,20]. 

Besides polymer chain relaxations, interfacial polarization typically appears at low *f* for the loss permittivity (*ε*′′) vs. *f* plots, and are caused by the sharp gradients in dielectric permittivity and/or charge conductivity across phase boundaries [21].

BDS examines interactions between alternating applied electric fields and polymer-affixed dipoles having reorientation mobility. The onset of long-range cooperative chain segmental motion, i.e., glass transition, as well as short range dipole rearrangements can be detected by BDS. 

In this study, we investigated the effect of blending chitosan with polyvinyl alcohol (PVA), followed by the insertion of AgNPs, on the dynamics of the secondary relaxations of chitosan. 

**Figure 2:**
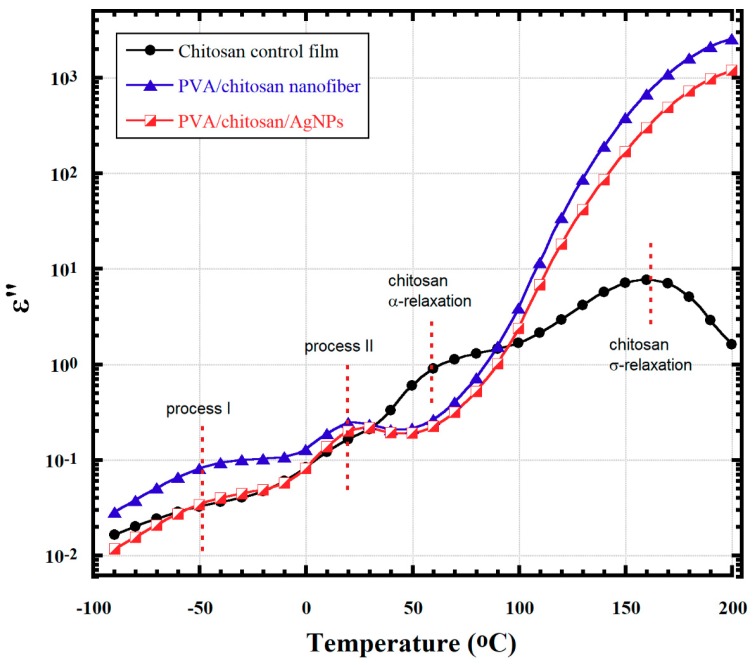
*ε*′′ vs. temperature at 1 kHz showing four different relaxation processes over a broad range of temperatures.

Figure 2 shows *ε*′′ vs. temperature curves at 1 kHz for the control chitosan film, its fiber blends with PVA, and a composite formed by AgNP insertion. Multiple relaxations are evident, although the frequency at which comparison is made can be as high as 1 kHz. High frequency decreases the time scale over which macromolecular motions can be sampled so that slower motions might be undetected. In the low temperature range, below 0 °C, a relaxation process was detected between −90 and −10 °C, which we call Process I. This process is not well resolved in the case of chitosan control and chitosan/PVA/AgNPs samples, while it appears to be very well defined in the case of the chitosan/PVA sample. Chitosan films were prepared by solvent casting in acetic acid solution, thus the final material appears to be highly protonated (contains NH_3_^+^ groups), behaving as an electrolyte and is highly sensitive to water. Many reports in the literature highlighted the presence of Process I in polysaccharides [22,23,24,25,26,27,28] and chitosan [29]. Process I was assigned to the local motions of the side groups in the repeating unit (–NH_2_, –NH_3_^+^, –OH, and –CH_2_OH) [16,29]. This process is of great interest for the current study and is discussed in detail below. Process II gets activated around −10 °C and we believe it is related to the local main chain segments’ motions that are facilitated by fluctuations within the glycosidic bonds. A higher degree of cooperativity not permissible for Process I is possessed in Process II [23]. 

The *third* relaxation for chitosan, with peak maximum around 60 °C, is thought to account for the long-range *α*-relaxation process, i.e., the glass transition temperature (*T_g_*) process according to Lazaridou and Biliaderis [28] and González-Campos et al. [29]. The peak position of this relaxation process is strongly dependent on both AgNPs content and the presence of water molecules, and could disappear upon heating close to 100 °C as water gets desorbed [29,30]. Water could actually get desorbed upon heating during the dielectric experiment and could affect the behavior of different relaxations, as reported in our study on Nafion polyelectrolyte membranes [31].

**Figure 3:**
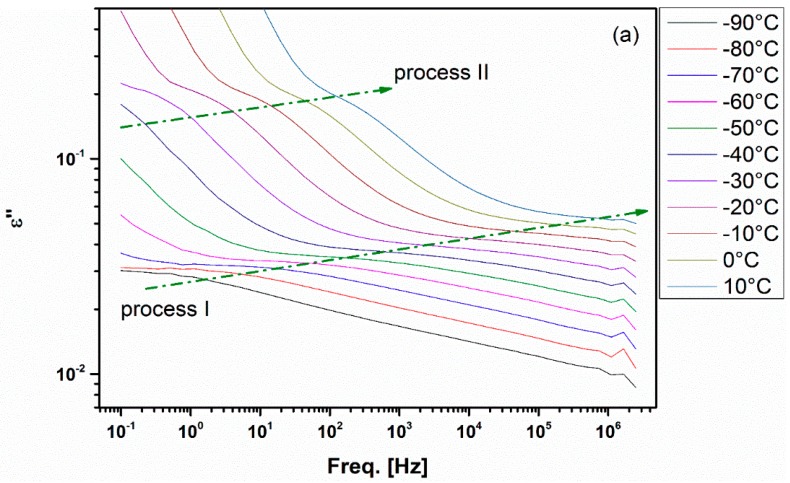

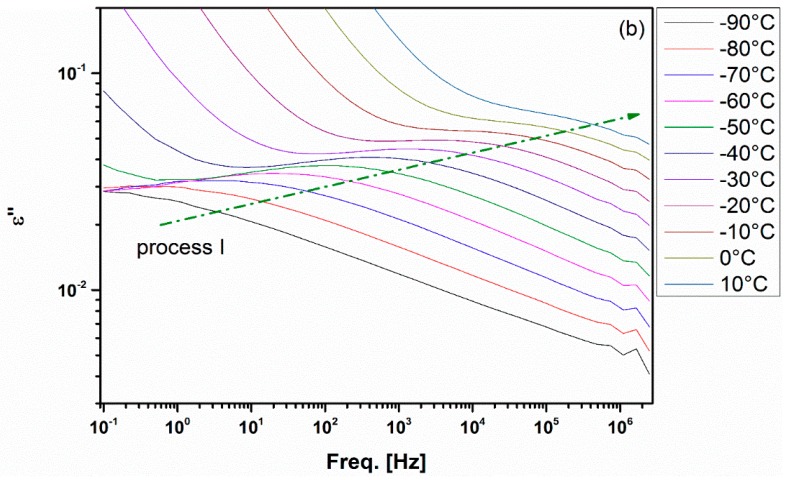
*ε***′′** vs. *f* spectra for the control chitosan film (**a**) and the electrospun chitosan/PVA/AgNPs composite blend (**b**) in the low temperature range. Colored arrows follow the progress of crests of different peaks with temperature.

The *fourth* relaxation for chitosan, with peak maximum around 160 °C, is attributed to the hopping of ions in the disordered structure of the biopolymers and is called *α*-relaxation [16,29,32]. These ions would be inadvertent impurities from catalysts that are used during the polymerization processes. The *α*-relaxation is reported to be independent of the moisture content in the sample [29]. It is important to mention that the *T_g_* for PVA is around 85 °C, depending on the degree of crystallinity and the amount of sorbed water [33]. In our case, as shown by the *ε*′′ vs. temperature spectra in Figure 3, it seems that PVA *T_g_* is greatly overwhelmed by the chitosan *σ*-relaxation. Possibly, this is due to the weak polarity of the PVA dipoles compared to those of the chitosan and the chitosan/AgNPs.

Figure 3 presents a further illustration of Processes I and II and their progress as temperature increases due to thermal activation. Process II gets activated at much lower temperature in the case of the chitosan sample which could be related to the effect of water molecules plasticization that are attached to the NH_3_^+^ and NH_2_ groups. Perhaps, this enhanced interaction of NH_3_^+^ side groups with water in the case of chitosan control sample is responsible for the fact that its Process I peak is ill-defined when compared to those for the chitosan/PVA and the chitosan/PVA/AgNPs samples (Figure 3) [16]. 

**Figure 4:**
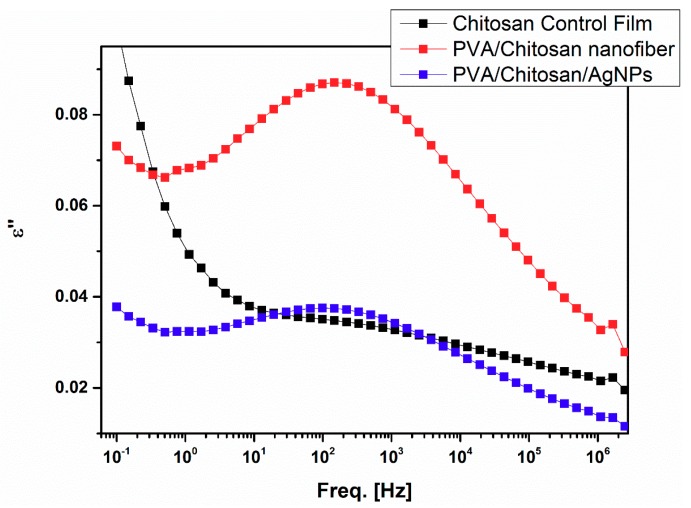
*ε*′′ vs. f spectra at −50 °C for the samples showing Process I.

Figure 4 shows a comparison of Process I’s peaks at −50 °C for all samples. The chitosan control peak intensity is strongly reduced when compared to those of the chitosan/PVA and the chitosan/PVA/AgNPs samples. This result confirms the above-mentioned conclusion that Process I gets strongly depleted, broadened, and less active with increased interactions of the NH_3_^+^ side groups with water. The peak intensity in the case of PVA/chitosan nanofiber sample is much higher compared to that of chitosan/PVA/AgNPs composite nanofiber. This could be due to the chitosan chain interaction with AgNPs making its dipoles less active. Peak maxima do not seem to shift with variable composition of the samples, although it is hard to be defined for the chitosan control sample. It is worth noting that the dielectric results could vary from a bulk film, chitosan control film, to a fiber samples, PVA/chitosan and chitosan/PVA/AgNPs. In this regard, the presence of air gaps, vs. compact dense film, and polymer chains orientation in the case of fiber samples could enhance the dielectric response of the sample, i.e., dielectric constant and peak intensity. However, the peak position could be more linked to interactions of the polymer chains with the surrounding environment, for example AgNPs. 

**Figure 5:**
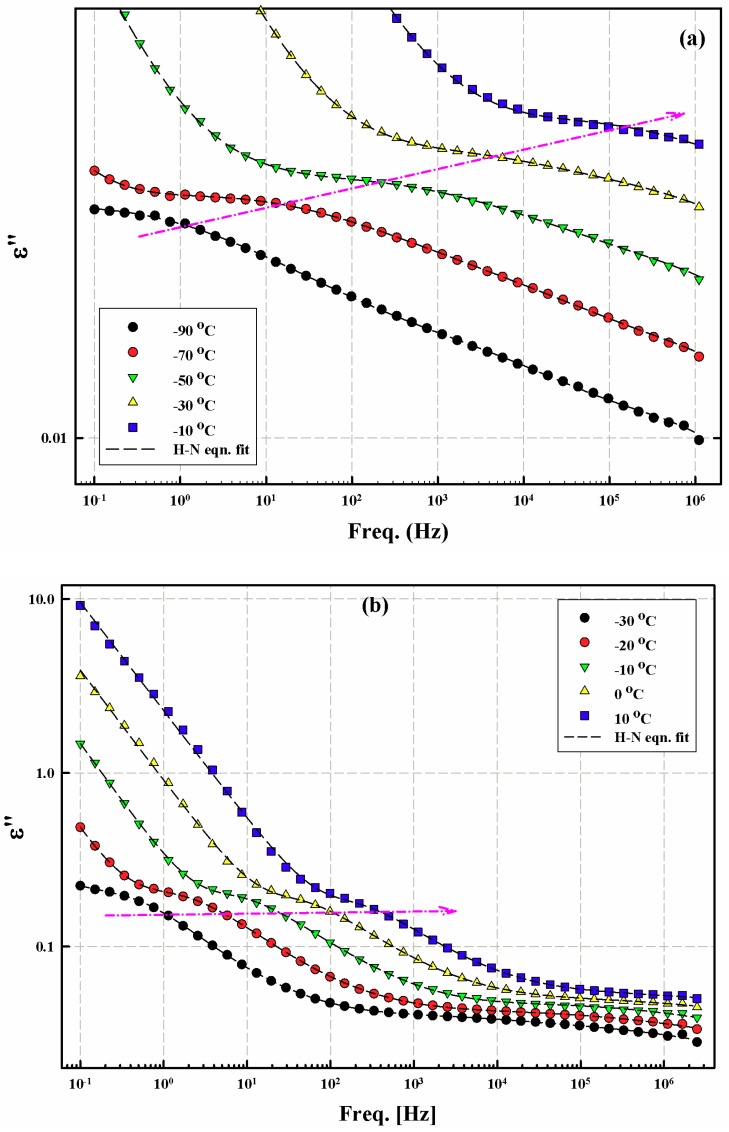
*ε*′′ vs. f spectra of the control chitosan film in the low temperature range showing: Process I (**a**); and Process II (**b**). Dashed lines represent the H-N equation fits to the spectra. Arrows indicate the progress of the relaxation peak crests with increased temperature.

Figure 5 shows the *ε*′′ vs. *f* spectra of the control chitosan sample after being fitted with the Havriliak–Negami (H-N) model (Equation (1)). Processes I and II relaxation peak maxima shift to higher frequencies as the temperature increases in a typical way. The dashed lines represent the H-N model fits to the spectra. All spectra appear to have a very good fit to the model. The linear monotonic uplift of *ε*′′ values proceeding to low *f*, after Process II activation, accounts for the dc conduction due to the presence of unintended ionic impurities, which could be left from the chitosan synthesis. It is also important to mention that hopping of the protonated amine groups between different monomer units along the chain could cause the same effect.

At low *f*, charge hopping events will have enough time to be sampled during the experimental time scale of a half period of oscillation (2*f*)^−1^ and before the applied electric filed reverses. Above *T_g_*, such hopping events will be even greater as they are facilitated by increased chain segmental mobility at the onset of the glass transition. The same fitting procedure was applied to all samples and the extracted shape parameters, *α_HN_* and *β_HN_*, for Process I relaxation peak are given in Table 1. According to a model by Schönhals and Schlosser [34], ${\left(\alpha_{HN}\right)}_{k}$ reveals the impact of intermolecular interactions of segments of *different* chains, whereas the ${\left(\alpha_{HN}\cdot \beta_{HN}\right)}_{k}$ parameter reflects intramolecular interactions between segments of a *single* chain, and they are linked to the *ε*′′ (*ω*) as follows:

(3) $$\varepsilon^{\prime \prime }\left(\omega \right)\;\sim \;\omega^{\alpha_{HN}}\;for\;(\omega \ll \omega_{{}^{\circ}})$$

(4) $$\varepsilon^{\prime \prime }\left(\omega \right)\;\sim \;\omega^{-\alpha_{HN}\cdot \beta_{HN}}\;for\;(\omega \gg \omega_{{}^{\circ}})$$

For Process I, which is well below the *T_g_* of chitosan and PVA, the environment of a chain unit differs locally due to the microstructure of the solid polymer and the heterogeneity of the side groups attached to each monomer unit [34]. Therefore, the diffusion process along the polymer chain seems to be greatly hindered as evidenced by the smaller values of the parameter ${\left(\alpha_{HN}\cdot \beta_{HN}\right)}_{k}$ (Table 1), which mostly lie in the range 0 < $-\alpha_{HN}\cdot \beta_{HN}$ < 0.5. On the other hand, small values of $\alpha_{HN}$ (<0.5) for all samples at all temperatures reflect the lack of large scale molecular motions of the chain units [34]. This is logically interpreted by the fact that Process I happens in the frozen state and also motions are hindered by presence of hydrogen bonding among side groups or through their interactions with AgNPs, as explained above. Overall, small values of the $\alpha_{HN}$ and $\beta_{HN}$ parameters could also reflect the broadening of the Process I peak [18], as shown in Figure 4. 

**Figure 6:**
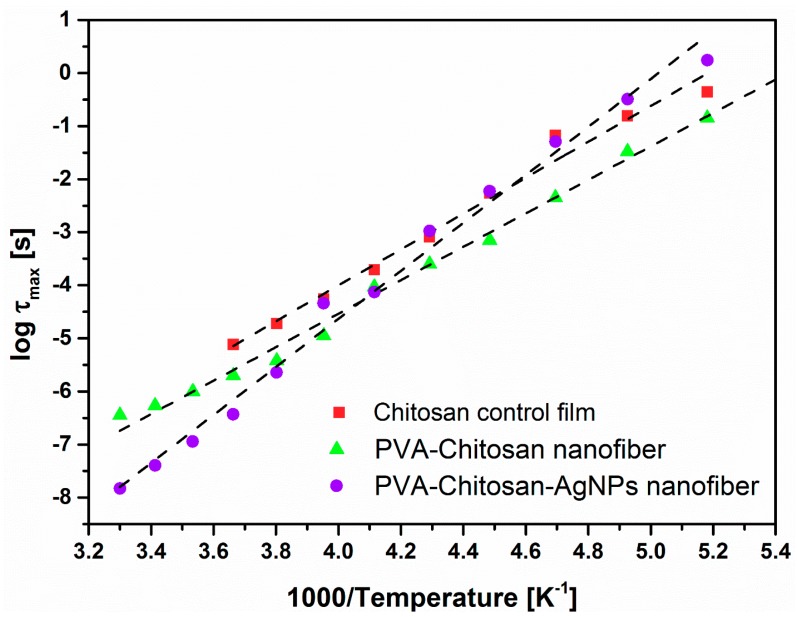
Arrhenius plots for the control chitosan film, electrospun chitosan/PVA and chitosan/PVA/AgNPs composite blends within the temperature range of Process I. Lines represent the best linear fits to the data.

The relaxation times *τ_max_* = 1/2*πf_max_* for Process I were extracted at each temperature for the spectra fitted with the H-N model. log *τ_max_*, when plotted against 1/*T* in Figure 6, shows strong Arrhenius behavior according to the equation:

(5) $$\tau_{max}(T)=\tau_{0}\exp\left(\frac{E_{a}}{RT}\right)\;$$

Values for activation energy, *E_a_*, for this relaxation are shown in Table 1.

**Figure 7:**
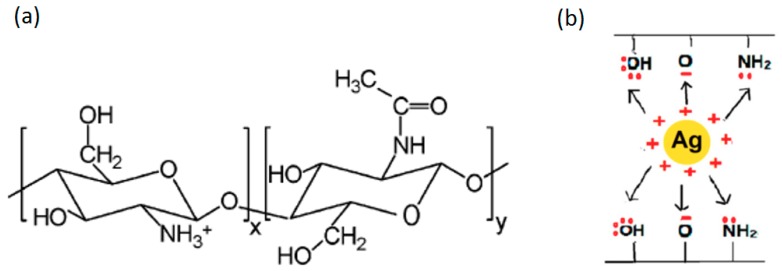
(**a**) The chitosan chemical structure after treatment with acetic acid; and (**b**) AgNPs interaction with negatively charged groups (NH_2_, O and OH) of chitosan [33].

The energy values in Table 2 are in the typical range for secondary relaxations in the glassy state of common polymers. Curves in Figure 6 are linear, rather than WLF-like, indicating that these motions are local. It is noted that the activation energy for the chitosan/PVA/AgNPs composite blend is much higher than that of the chitosan control film. This supports our earlier conclusion of the existence of strong interactions between the amine groups and the AgNPs, as depicted in Figure 7 and reported by Hadipour-Goudarzi et al. [35]. 

### 3.3. Kramers–Krönig Integral Transformation for the Dielectric Spectra in the Chitosan Glass Transition Region 

The Kramers–Krönig (KK) equation has two terms, the first is an Ohmic resistance term (similar to the one in HN equation) and the second term is an integral transformation from the real to the imaginary permittivity assessed at *ω*_0_, labeled *ε*′′*_kk_* [36,37]:

(6) $$\varepsilon_{}^{\prime \prime }(\omega_{0})=\frac{\sigma_{0}}{\varepsilon_{O}\omega_{0}}+\frac{2}{\pi }\int_{0}^{\infty }\varepsilon^{\prime }(\omega )\frac{\omega }{\omega^{2}-\omega_{0}^{2}}d\omega =\frac{\sigma_{0}}{\varepsilon_{O}\omega_{0}}+\varepsilon_{kk}^{\prime \prime }\;$$

As discussed for Equation (1), omitting the obscuring dc conduction term from measured values of *ε*′′(*ω*_0_) causes the relaxation terms in the sum to become more distinctive. Equation (6) presents another method to this correction for conductivity due to the fact that the integral *ε*′′*_kk_* contains only the real permittivity, which does not include dc conductance. Therefore, pure chain relaxations can be extracted from the experimental *ε*′ values over a wide frequency range and through using a numerical integration that yields *ε*′′*_kk_*. 

**Figure 8:**
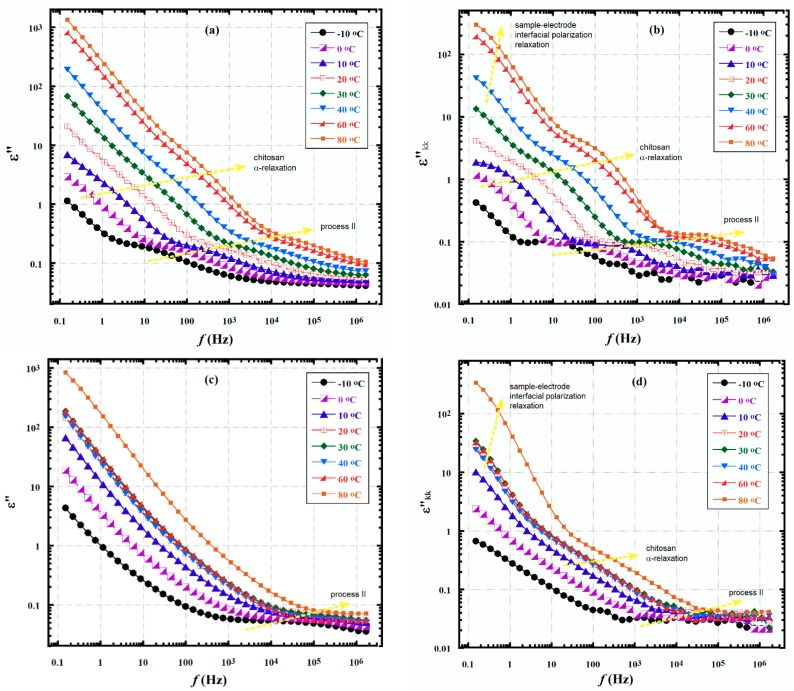
*ε*′′ vs. *f* spectra for control chitosan film (**a**,**b**) and the electrospun chitosan/PVA/AgNPs composite blend (**c**,**d**) before and after the KK transformation, in the temperature range of the chitosan *T_g_*. Dashed arrows indicate the progress of different relaxations with increased temperature.

KK transformations were calculated using a software developed based on an algorithm provided by Steeman and van Turnhout. The software was developed using the C language and runs under a Linux operating system. More details about this transformation can be found in References [36,37]. *ε*′′*_kk_* values were calculated for the samples from experimental *ε*′(*ω*) data for temperatures within the chitosan *T_g_* using this program, and results are exhibited in Figure 8. The resolution of this chitosan *α*-relaxation peak is enhanced and its change with temperature appears much clearer than it was before the KK transformation. The *T_g_*-related peak at high *f* shifted to the right as temperature is increased, as expected. The slight downward bend of the graphs at low *f* (Figure 8b,d) is tentatively assumed to be due to the sample–electrode interfacial polarization relaxation process [38,39,40]. This proposition is reinforced by analogous high ε′ values at low *f* (figure not shown). This feature monotonically shifts upward with increasing temperature which can be elucidated by the fact that the accumulated charges in the near-electrode regions gain more mobility causing greater positive–negative charge separation, especially when the chain segments are more mobile at *T* > *T_g_* and charge hopping is easy [41]. It has been previously reported that, “It is important to note that the Kramers-Krönig transformation is model-independent, and, as such, is not linked to the dipole rotation mechanism as it is in the Debye model and its subsequent improvements and variations. In short, it does not require a molecular underpinning and does not impart a bias in data transformation” [42].

## 4. Conclusions

Broadband dielectric spectroscopy was used to interrogate the macromolecular motions in chitosan and its nanofiber blend with PVA and their composite nanofiber with AgNPs. The dynamics of low temperature relaxations were analyzed by fitting the Havriliak–Negami equation to the dielectric spectra. Two major processes were evidenced in the spectra, namely Processes I and II. Process I was related to the local motions of the side groups in the repeat units of the chitosan chains, while Process II was assigned to the local chain segments motions facilitated by glycosidic bonds fluctuations. Activation energy values were calculated from the Arrhenius plots and interactions of the AgNPs with amine side groups of the chitosan chains were evidenced. Linear response of the Arrhenius plots for Process I support the assignment of the local short-scale nature of that process. Additionally, activation energy for the chitosan/PVA/AgNPs composite blend was much higher than that of the chitosan control film, which supports the existence of strong interactions between the amine groups in chitosan and the AgNPs. The local nature and broadness of Process I was also supported by the fact the shape parameters values for this relaxation peak were generally smaller than 0.5.

The Kramers–Krönig integral transformation was used to extract dc-free *ε*′′ vs. *f* relaxation peaks from experimental *ε*′ vs. *f* data, in the region of the chitosan *T_g_*. This mathematical procedure excludes the necessity to directly subtract a dc conductivity influence from experimental *ε*′′ vs. *f* curves as with the H-N equation. The K-K transformation resulted in well-resolved α-relaxation peaks for the chitosan in the electrospun nanocomposite samples. 

Broadband dielectric spectroscopy is a valuable tool to analyze the long- and short-ranged motions and thermal transitions over a range of temperatures in these composites blends. This powerful technique proves to provide very important information regarding the AgNPs behavior with the polymer matrix around them, which would help understand their performance in different biomedical applications.

## Figures and Tables

**Table 1:** Relaxation peak shape parameters extracted from the H-N model fitting to Process I spectra for all samples at different temperatures.

Temperature (°C)	Chitosan Control Film			PVA/Chitosan Nanofiber			PVA/Chitosan/AgNPs Nanofibers		
	*β_HN_*	*α_HN_*	MSD *	*β_HN_*	*α_HN_*	MSD	*β_HN_*	*α_HN_*	MSD
−90	1.00	0.23	1.3034 × 10^−4^	0.40	0.41	4.7535 × 10^−4^	0.30	0.43	1.4057 × 10^−4^
−80	1.00	0.24	2.2850 × 10^−4^	0.38	0.38	5.2658 × 10^−4^	0.36	0.38	1.5265 × 10^−4^
−70	1.00	0.25	2.2410 × 10^−4^	1.00	0.41	6.4542 × 10^−4^	0.47	0.32	1.9950 × 10^−4^
−60	1.00	0.25	2.5342 × 10^−4^	0.52	0.32	5.2232 × 10^−4^	0.50	0.33	1.6653 × 10^−4^
−50	1.00	0.26	3.2416 × 10^−4^	0.64	0.31	6.3177 × 10^−4^	0.49	0.37	1.9142 × 10^−4^
−40	0.71	0.41	1.2279 × 10^−3^	0.61	0.32	6.3736 × 10^−4^	0.69	0.32	2.0201 × 10^−4^
−30	0.70	0.41	3.9269 × 10^−4^	0.82	0.29	1.0297 × 10^−3^	0.69	0.29	8.3479 × 10^−4^
−20	0.51	0.26	4.8154 × 10^−4^	0.97	0.29	5.5235 × 10^−3^	0.89	0.30	1.0103 × 10^−2^
−10	0.25	0.40	3.2322 × 10^−3^	0.72	0.40	1.0744 × 10^−1^	0.31	0.51	1.5459 × 10^−2^
0	0.27	0.33	5.3110 × 10^−2^	-	-	-	0.90	0.32	1.5474 × 10^−2^
10	0.27	0.33	1.0597 × 10^−1^	-	-	-	0.89	0.32	1.4379 × 10^−1^
20	0.24	0.35	4.2959 × 10^−1^	-	-	-	0.51	0.43	1.3868 × 10^−2^

* MSD is the mean standard deviation of the fit parameters.

**Table 2:** Calculated activation energies for Process I relaxations of all samples.

Sample	Activation Energy (kJ/mol)	Fit R^2^	Pre-Exponential Factor (*τ*_ο_)
Chitosan control film	64.7	0.98	2.93 × 10^−18^
PVA-chitosan nanofiber	60.3	0.99	7.15 × 10^−18^
PVA-chitosan-AgNPs nanofiber	86.5	0.99	1.84 × 10^−23^
